# The Influence of Wire Speed on Phase Transitions and Residual Stress in Single Crystal Silicon Wafers Sawn by Resin Bonded Diamond Wire Saw

**DOI:** 10.3390/mi12040429

**Published:** 2021-04-14

**Authors:** Tengyun Liu, Peiqi Ge, Wenbo Bi

**Affiliations:** 1 School of Mechanical & Automotive Engineering, Qilu University of Technology, Jinan 250353, China; lty198614@163.com; 2 School of Mechanical Engineering, Shandong University, Jinan 250061, China; biwenbo@sdu.edu.cn; 3 Key Laboratory of High-Efficiency and Clean Mechanical Manufacture at Shandong University, Ministry of Education, Jinan 250061, China

**Keywords:** diamond wire saw, silicon wafer, phase transition, residual stress, wire speed

## Abstract

Lower warp is required for the single crystal silicon wafers sawn by a fixed diamond wire saw with the thinness of a silicon wafer. The residual stress in the surface layer of the silicon wafer is the primary reason for warp, which is generated by the phase transitions, elastic-plastic deformation, and non-uniform distribution of thermal energy during wire sawing. In this paper, an experiment of multi-wire sawing single crystal silicon is carried out, and the Raman spectra technique is used to detect the phase transitions and residual stress in the surface layer of the silicon wafers. Three different wire speeds are used to study the effect of wire speed on phase transition and residual stress of the silicon wafers. The experimental results indicate that amorphous silicon is generated during resin bonded diamond wire sawing, of which the Raman peaks are at 178.9 cm^−1^ and 468.5 cm^−1^. The ratio of the amorphous silicon surface area and the surface area of a single crystal silicon, and the depth of amorphous silicon layer increases with the increasing of wire speed. This indicates that more amorphous silicon is generated. There is both compressive stress and tensile stress on the surface layer of the silicon wafer. The residual tensile stress is between 0 and 200 MPa, and the compressive stress is between 0 and 300 MPa for the experimental results of this paper. Moreover, the residual stress increases with the increase of wire speed, indicating more amorphous silicon generated as well.

## 1. Introduction

Fixed diamond wire sawing, a novel slicing technology, is used to process hard and brittle materials, such as single crystal silicon, polycrystalline silicon, silicon carbide, sapphire, and so on. Up to now, fixed diamond wire sawing occupies more than 90% of the wafering technology for single crystal silicon [1]. To minimize the material loss, the diameter of the diamond wire is becoming smaller and smaller. According to an International Technology Roadmap for Photovoltaic (ITRPV) report, the kerf loss for diamond wire sawing was about 80 μm in 2018. The thickness of the silicon wafer becomes smaller and smaller, requiring higher wire sawing quality.

The fixed diamond wire saw can be grouped into two kinds of wire saws: the electroplated diamond wire saw, and the resin boned diamond wire saw. Their sawing performances are different. According to the experimental results from the reference, the resin bonded diamond wire saw has a better sawing quality compared to the electroplated diamond wire saw [2]. This is not only because the homogeneity of protrusion heights for the diamond abrasive is much better, but also the resin layer can absorb a certain impact energy induced by wire movement. Thus, better wire sawing quality can be obtained by using a resin bonded diamond wire saw. Based on this point, the resin bonded diamond wire saw is used in this paper.

Residual stress is the main factor leading to the warpage of as-sawn wafers. As a kind of internal stress, the residual stress is generated by the material removal, deformation, and uneven volume changes of the material [3]. For the process of diamond wire sawing, residual stress is induced by the interaction of elastic-plastic deformation, processing heat and phase transitions [4]. The value and distribution of residual stress mainly depends on the phase transitions and elastic-plastic deformation. Therefore, the influence of the sawing process parameters on the residual stress is required to be analyzed. It is important to optimize the sawing process to obtain thinner silicon wafers with lower warpage. 

The material removal process for a resin bonded diamond wire saw is the movement of the diamond abrasives’ squeeze, scratch and plough on the silicon surface. Diamond abrasives generate a large hydrostatic pressure on the surface of the silicon wafers. Single crystal silicon is a kind of pressure-sensitive material, which is very unstable under high pressure and undergoes phase transition. That is, the single crystal silicon structure changes under pressure. Silicon structure mainly changes from Si-I with a diamond structure to Si-II with a β-sn phase, Si-V with a simple hexagonal structure and Si-VII with a close-packed hexagonal crystal structure. After the pressure is released, it changes from Si-II, etc. to Si-III with a body-centered cubic structure, amorphous silicon, etc. Many scholars have studied the pressure value during the phase transition of single crystal silicon. As the pressure increases, the diamond-structured silicon first transforms into Si-II with the β-sn phase when the pressure reaches 10–12 GPa. When the pressure reaches about 16 GPa, the single crystal silicon transforms from Si-II to Si-V. When the pressure reaches about 37.6–42 GPa, Si-V will transform into Si-VII. When it reaches 78 GPa, Si-VII will transform into a face-centered cubic crystal phase. When the pressure disappears, the Si-II will not return to the diamond phase, but will transform into Si-III, Si-VIII, amorphous silicon, etc.

Regarding the study of phase transitions and residual stress in single crystal silicon wafers produced by sawing, there are more scholars who focus on the study of phase transitions, while only fewer scholars have studied the residual stress [5,6,7,8,9,10]. Würzner et al. studied the residual stress on the surface layer of silicon wafers sawn by fixed diamond wire sawing using Raman characterization. Their experimental results show that higher wire speed leads to lower residual stress, but with more amorphous silicon formation [11]. Yang et al. used the infrared polariscope method to measure the residual stress on the surface of the full-size wafer, and studied the effects of residual stress and surface defects on the fracture strength of silicon wafers [12]. Banerjee et al. analyzed the particulate swarf and fibrillar swarf generated by the diamond-coated wire sawing of silicon ingot, in order to obtain the stress during wire sawing. Their experimental results indicate that residual tensile stress is liable to occur in the condition of a ductile cutting regime. On the contrary, residual compressive stress is easily generated in the condition of a brittle cutting regime [13]. Wu studied the residual stress using the method of Raman spectroscopy for the wafers sawn by slurry wire sawing and fixed diamond wire sawing. The experimental results show that the as-sawn wafers reveal a noticeable vertical stress pattern along the wire feed direction [14]. Furthermore, Popovich et al. studied the relationship between the residual stress on polycrystalline silicon and the microstructure, defects and processing conditions. Their research results show that the residual stress on the grain boundary is larger by about 50~70 MPa than that on another region. The effect of phase transitions on the mechanical properties of silicon wafers is studied also [15]. There are few researches that focus on the residual stress of silicon wafers sawn by a fixed diamond wire saw. Besides that, the researches on the effects of processing parameters on residual stress and phase transitions are insufficient and further research is needed. 

In this paper, the relationship between Raman spectrum shift and the depth of the amorphous silicon layer and residual stress is established. Based on this relationship, the depth of the amorphous silicon layer and residual stress for silicon wafers sawn by a resin bonded diamond wire saw are obtained by measuring Raman spectroscopy. This paper provides a new method to research the slicing mechanism of a diamond wire saw. In addition, this article focuses on the influence of wire speed on the residual stress and the depth of the amorphous silicon layer. The experimental results are of great significance for revealing the slicing mechanism and the warping deformation of silicon wafers.

## 2. Materials and Methods

### 2.1. Resion Bonded Diamond Wire Saw

#### 2.1.1. Multi-Wire Saw Machine

**Figure 1:**
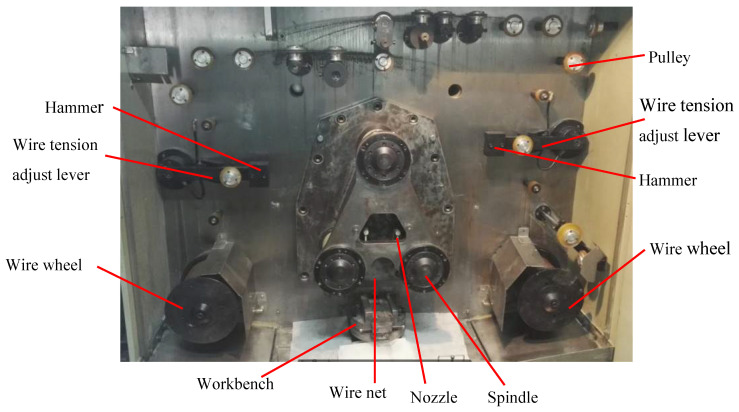
The structure of multi-wire sawing machine.

The type of the multi-wire saw machine used in this experiment is the YJXQ120B multi-wire saw machine. Its structure is shown in Figure 1. The resin bonded diamond wire saw is wrapped around on the three spindles, and the wire net is formed. The diamond wire is forced by the three spindles to do the reciprocating motion. The single crystal silicon ingot is fed upward along the direction vertical to the wire net during wire sawing, and the cutting fluid is sprayed from the nozzle to realize the lubricating and cooling function. The wire tension is adjusted by two levers, which are controlled by gas-sealed bearing. One gravity hammer is installed at the end of the lever, the weight of which can be adjusted to realize the adjustment of the wire tension. The maximum size of work piece that can be sliced is 120 mm × 120 mm × 75 mm. The maximum reciprocating motion velocity for the diamond wire is 280 m/min, and the feed speed can be adjusted between 0.1 mm/h and 100 mm/h.

#### 2.1.2. Resin Bonded Diamond Wire Saw

**Figure 2:**
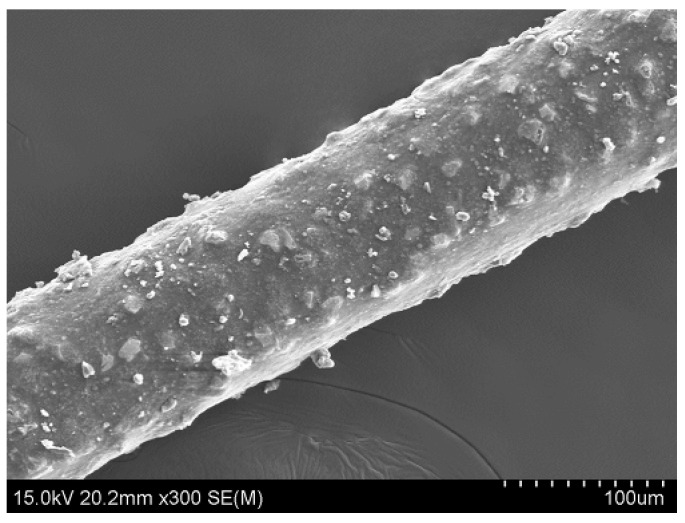
The morphology of resin bonded diamond wire saw.

The resin bonded diamond wire saw used in this experiment is bought from one vendor, and its morphology is shown in Figure 2. The diameter of this kind of diamond wire is between 95 μm and 105 μm, and the size of the diamond abrasive is between 8 μm and 16 μm. The abrasive density is 1200 grits/mm^2^. The tensile strength of the diamond wire is 2546 MPa.

The basic solution of cutting fluid used in the experiment is bought from one retailer, of which the value of PH is about 7~8, and its viscosity is between 90 m^2^/s and 130 m^2^/s. The water is mixed into the basic solution according to the proportion of 1:400 to obtain the cutting liquid.

**Figure 3:**
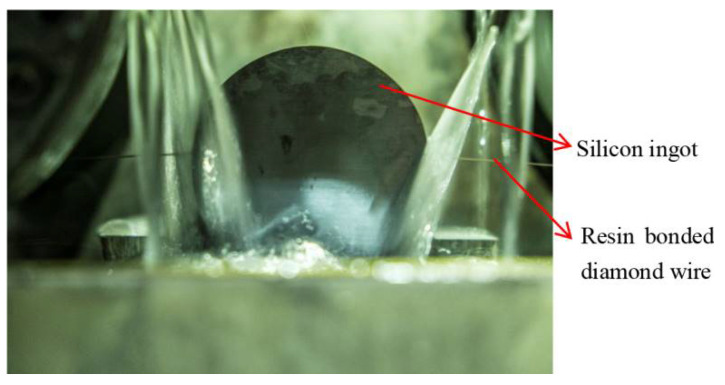
The process of resin bonded diamond wire sawing silicon ingot.

The silicon ingot used in this experiment is a P-type (111) silicon ingot, of which the diameter is 56 mm. The slicing direction is (1, −1, 0).Three sawing experiments have been done according to different wire speeds, and the experimental parameters are shown in Table 1. Three wire sawing experiments have been done by using the above three processing parameters. The silicon ingot is adhered to the workbench by adhesive and is fed along the direction vertical to the wire net. The process of resin bonded diamond wire sawing is shown in Figure 3. 

### 2.2. Raman Spectrum Characterization

After wire sawing, three kinds of as-sawn silicon wafer are obtained, and they are characterized by the Microscope Raman spectrometer. The spectrometer sensitivity and distinguishability are ±0.2 cm^−1^ and 585 nm respectively. An excitation laser with a wavelength of 633 nm and a resulting laser power of 1.23 mW are used to get the Raman mappings. Besides that, the scan range is 100–700 cm^−1^.

**Figure 4:**
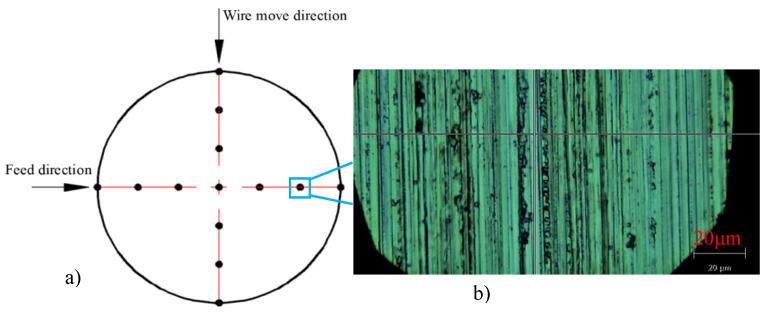
Schematic diagram of distribution for characterized points and the sliced surface topography. (**a**) Distribution of characterized points; (**b**) the sliced surface topography of silicon wafer.

Thirteen points are detected along the feed direction and wire movement direction. The distribution of these detective points is shown in Figure 4. The Raman spectrum of single crystal silicon under the nature state has a sharp and fully symmetrical phonon band peak with a wave number of 521 cm^−1^. The tensile stress causes the movement of the Raman peak to lower the wave number, while the compressive stress leads to the Raman peak moving to the direction of a higher wave number. The peak shifts were calibrated to a stress value according to Reference [16]: a Raman shift of ±3.2 cm^−1^ corresponds to ±1 GPa stress. Thus, the stress value can be obtained by the Raman shift, and the corresponding residual stress distribution can be obtained.

## 3. Results

### 3.1. Phase Transition

**Figure 5:**
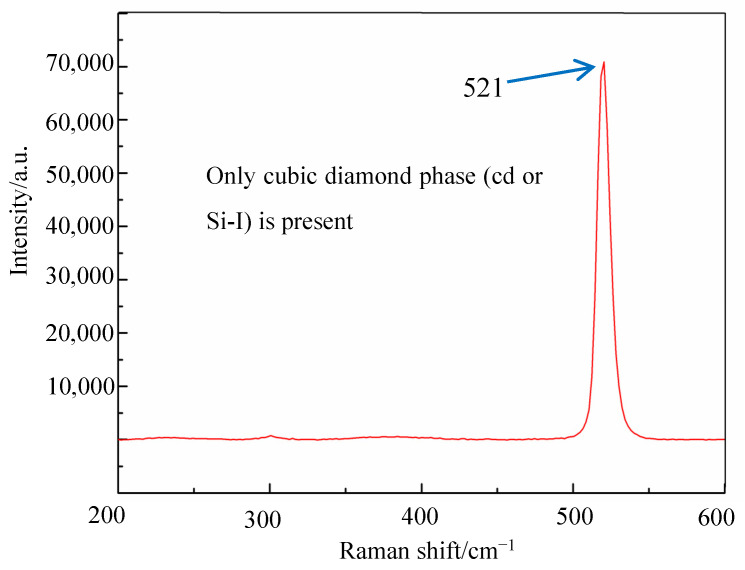
The Raman spectrum of single crystal silicon wafers.

A high contact pressure is generated during diamond wire sawing between the diamond abrasive and the work piece, leading to the phase transitions. The structure of single crystal silicon changes into an amorphous structure, showing different Raman peaks in the Raman spectrum. The typical Raman shift of Raman peaks for amorphous silicon are 150 cm^−1^, 380 cm^−1^, 430 cm^−1^ and 470 cm^−1^ based on the experimental results of reference [17]. The Raman peak of single crystal silicon in the state of nature is 521 cm^−1^, which is shown in Figure 5.

**Figure 6:**
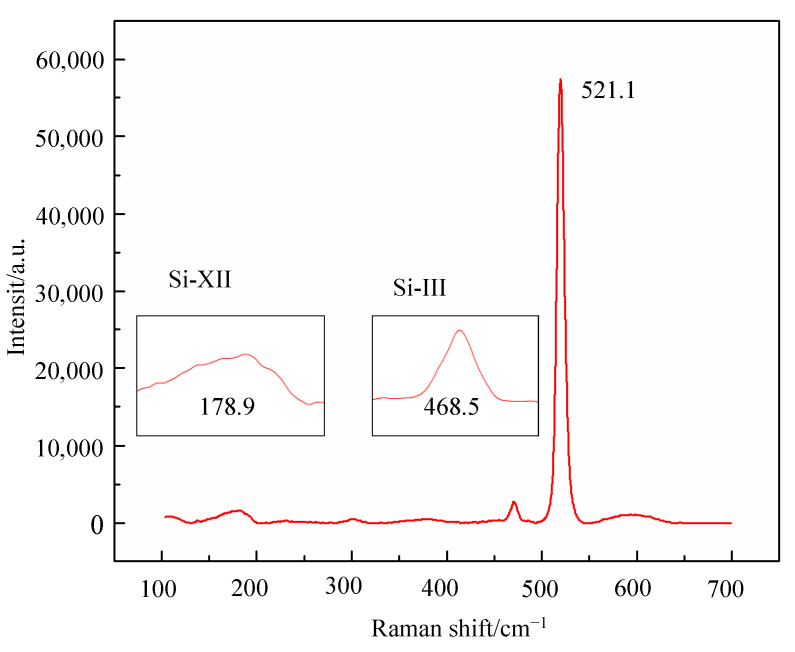
The Raman spectrum for the as-swan silicon wafer.

The Raman spectrum for the as-sawn silicon wafer is shown in Figure 6. Because of the residual stress on the surface layer, the peak corresponding to the Raman shift is not 521 cm^−1^, but is 521.1 cm^−1^ which is a little larger. In addition, the Raman peaks at about 178.9 cm^−1^ and 468.5 cm^−1^ also exist, which indicates that Si-XII and Si-III exist on the wafer surface layer. In general, the major component in the silicon wafer surface layer is single crystal silicon, and the other component is amorphous silicon.

According to the study of Reference [7], the depth of the amorphous silicon layer can be obtained by the ratio *r*, which is the ratio of the amorphous surface area and the un-phase transitions surface area. The other method for getting the ratio *r* is by calculating the surface of under the Raman spectrum curve of a different peak value. After obtaining *r*, Equations (1) and (2) are used to get the amorphous layer depth. The Raman spectrum should be fitted first. The peak height *h_a_* (a.u) and bandwidth *b_a_* ( cm^−1^) for amorphous silicon, and the peak height *h_c_* (a.u) and bandwidth *b_c_* ( cm^−1^) for single crystal silicon can be obtained by the above curve fitting method.

(1) $$r=\frac{h_{a}b_{a}}{h_{c}b_{c}}\frac{1}{\sqrt{\pi }}$$

(2) $$d_{a}=33.3\ln\left(\frac{8.84+15r}{8.84+0.167r}\right)$$

**Figure 7:**
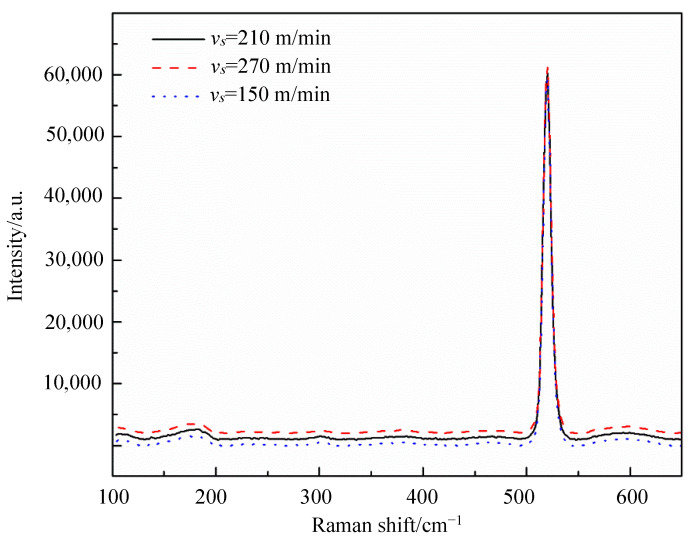
The Raman spectrum of wafers sawn by different wire speeds.

The ratio *r* and depth of the amorphous silicon layer *d_a_* (nm) for the three kinds of as-sawn silicon wafers are shown in Table 2. It can be seen that the ratio *r* and the depth of the amorphous silicon layer *d_a_* increase with the increasing of the wire speed. This indicates that more amorphous silicon is generated with the increase of the wire speed. This phenomenon agrees with the changes of Raman intensity of these three kinds of sawn wafers, as shown in Figure 7. It can be seen that the Raman intensity increases with the increase of the wire speed. The higher the Raman intensity, the more amorphous silicon is generated, thus the phase transition area is increased, leading to a larger ratio *r* and a larger amorphous silicon layer depth.

Based on the above experimental results, it can be said that more phase transitions are generated with the increase of the wire speed. The reason is that more diamond abrasives take part into slicing and the cutting force decreases with the increase of wire speed. Thus, the average cutting force on a single abrasive decreases, resulting in a smaller cutting depth and more diamond abrasives remove material in the ductile regime mode. Therefore, more amorphous silicon is formed with the increase of the wire speed.

### 3.2. Residual Stress

#### 3.2.1. The Distribution of Residual Stress

For each silicon wafer, 13 points are characterized by using the above measurement method. The Raman spectrum at each point is measured to obtain the Raman shift by subtracting the number of Raman shifts of a single crystal silicon. A Raman shift of ±3.2 cm^−1^ corresponds to ±1 GPa stress. Thus, the stress value can be obtained by the Raman shift. If the Raman shift is a negative value, the residual stress is tensile stress. On the contrary, the residual stress is compressive stress. 

**Figure 8:**
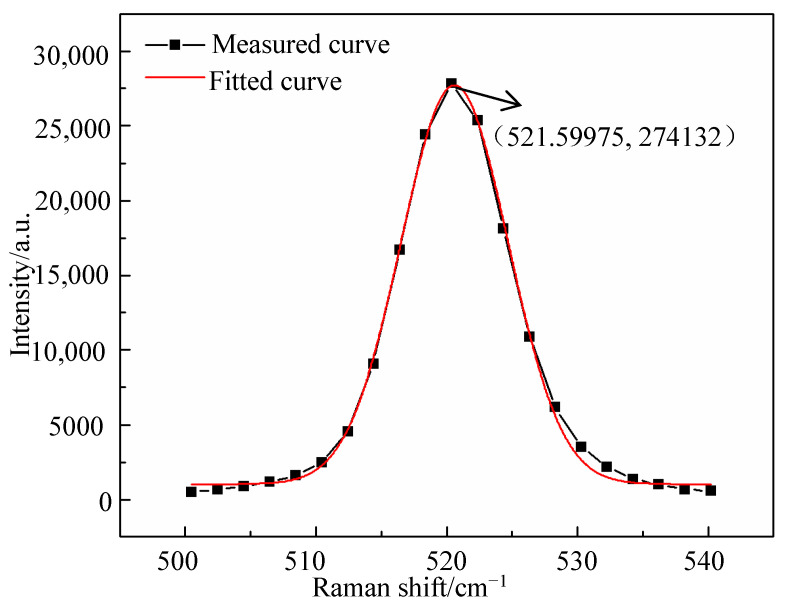
The fitted Raman curve for the Raman peak.

Usually, Lorentzian analysis is used for crystal peaks, and Gaussian analysis is more suitable for amorphous peaks. Therefore, the Gaussian fitting method is used in this paper. For example, Figure 8 is the fitted Raman curve and measured curve. The R-square coefficient is 0.98835, which is close to 1, indicating the fitting function has a good fitting degree. It can be seen that the fitted value of the Raman peak is 521.59975 cm^−1^, which is larger than 521 cm^−1^, indicating that compressive stress exists at this point.

**Figure 9:**
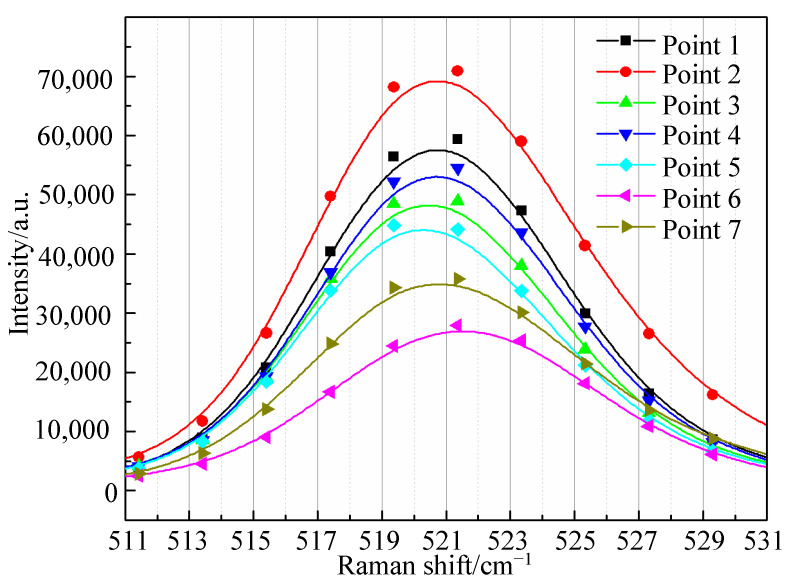
Seven fitted Raman peak curves for one kind of wafer.

**Figure 10:**
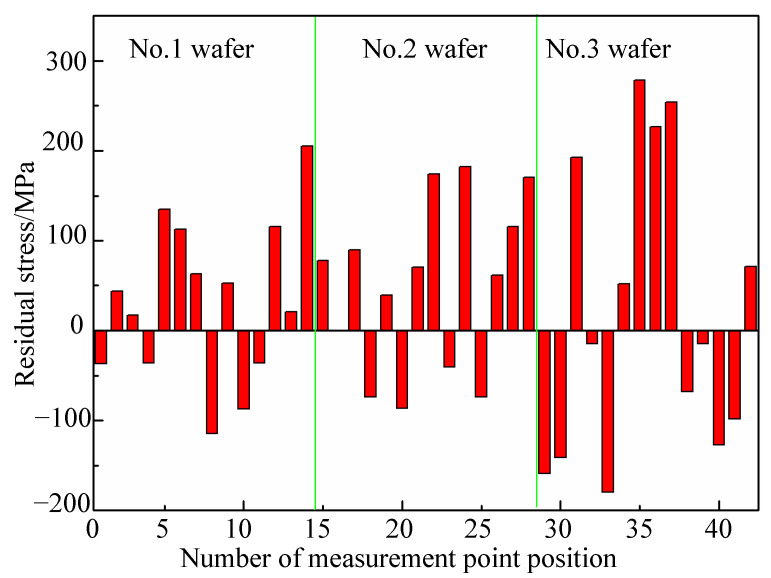
The residual stress of three silicon wafers.

Three kinds of silicon wafers are measured by this above method. The Raman peak and Raman shift of seven measurement points on one as-sawn silicon wafer are shown in Figure 9. Thirteen points are measured on each wafer shown in Figure 4. The residual stress and Raman shift for these measurement points are shown in Figure 9 and Figure 10. It can be seen that the residual stress on these three kinds of wafers distribute unevenly. The tensile stress and compressive stress exist simultaneously. 

In Figure 10, the negative value indicates the residual stress is tensile stress. Conversely, the positive value is compressive stress. It can be seen that the residual tensile stress is between 0 and 200 MPa, while the compressive stress is between 0 and 300 MPa for the experimental results of this paper. In addition, the number of points demonstrating residual compressive stress is a little larger than those demonstrating residual tensile stress, the numbers of which are 24 and 18 respectively. The average tensile stress is smaller than the average compressive stress.

There is both tensile stress and compressive stress on the sliced single crystal silicon wafer surface layer. The residual stress on the sliced surface layer is generated due to the phase transitions, cutting heat and the elastic-plastic deformation during the diamond wire sawing. The value of the residual stress depends on the combined effect of these above three factors. The cutting force during diamond wire sawing can be resolved into normal cutting force and tangential cutting force. The normal force presses the diamond abrasive into the material and determines the cutting depth of the diamond abrasive during diamond wire sawing. The tangential cutting force drives the diamond abrasives scratching on the material surface, realizing the material removal. Therefore, the material undergoes tensile deformation, and then some of the tensile deformation is restored after the material fracture. Besides that, the pressing action leads to a large residual compressive stress on the slicing surface. Hence, the mixed stress state of residual tensile stress and compressive stress exist on the wafer surface sawn by a fixed diamond wire saw.

#### 3.2.2. The Effect of Wire Speed on Residual Stress

**Figure 11:**
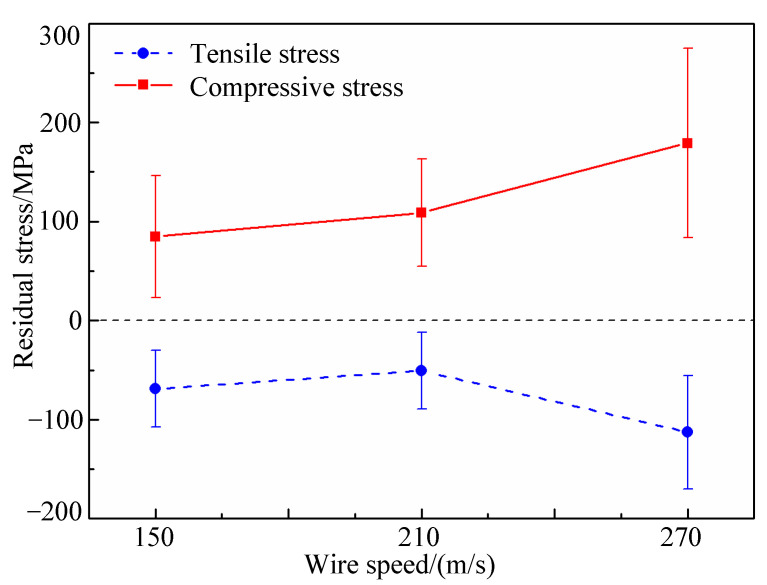
The change of residual stress with the increase of wire speed.

The average residual compressive stress and tensile stress for the three kinds wafers processed by different wire speeds are shown in Figure 11. It can be seen that, the average values of residual compressive stress for the no. 1 silicon wafer, no. 2 wafer and no. 3 wafer are 84.972 MPa, 108.945 MPa and 179.183 MPa respectively. The average values of residual tensile stress for no. 1 wafer, no. 2 wafer and no. 3 wafer are −68.941 MPa, −50.534 MPa and −112.71 MPa respectively. The residual compressive stress increases with the increase of the wire speed, and the tensile stress increases slightly with the increase of the wire speed.

According to the previous analysis, the cutting force decreases with the increase of the wire speed. Thus, more diamond abrasives remove material in ductile mode, and more amorphous silicon generates on the sliced surface. The phase transitions make the stress inside of the material and cannot be released, resulting in a higher stress gradient. Hence, the residual stress increases with the wire speed. This is in accord with the experimental results in Table 2.

## 4. Conclusions

This paper studies the phase transitions and the residual stress in the surface layer of silicon wafers sawn by a resin bonded diamond wire saw. The Raman spectroscopy method is used to analyze the phase transitions and residual stress. The influences of the wire speed on the phase transitions and residual stress are analyzed. Based on the experimental results of this paper, the following conclusions can be obtained: 

(1) There is amorphous silicon in the single crystal silicon wafer surface layer sliced by a resin bonded diamond wire saw, of which the Raman shifts are 178.9 cm^−1^ and 468.5 cm^−1^.

(2) There is both compressive stress and tensile stress on the silicon wafer surface layer. The residual tensile stress is between 0 and 200 MPa, and the compressive stress is between 0 and 300 MPa for the experimental results of this paper.

(3) The residual stress and the depth of the amorphous silicon layer increases with the increase of the wire speed, which indicates more amorphous silicon is generated with the increase of the wire speed.

## Figures and Tables

**Table 1:** The processing parameters of wire sawing experiment.

No.	Wire Seed	Feed Speed	Wire Tension
1	150 m/min	0.2 mm/min	12 N
2	210 m/min	0.2 mm/min	12 N
3	270 m/min	0.2 mm/min	12 N

**Table 2:** The ratio r and depth of amorphous silicon layer for three kinds of sawn silicon wafers.

Wire Speed	Raman Intensity Ratio *r*	Depth of Amorphous Layer/nm
*v_s_* = 150 m/min	0.13	6.556
*v_s_* = 210 m/min	0.27	12.39
*v_s_* = 270 m/min	0.59	22.73

## Data Availability

The data presented in this study are available on request from the corresponding author.
